# Allergic diseases aggravate the symptoms of SARS-CoV-2 infection in China

**DOI:** 10.3389/fimmu.2023.1284047

**Published:** 2023-12-22

**Authors:** Huishan Zhang, Jilei Lin, Jinhong Wu, Jing Zhang, Lei Zhang, Shuhua Yuan, Jiande Chen, Qiuyu Tang, Ailian Zhang, Yuxia Cui, Xiaojuan Xu, Hongxie Dai, Hongbo Shi, Xiaowei Hu, Dan Xie, Jing Chen, Fengquan He, Yong Yin

**Affiliations:** ^1^ Department of Respiratory Medicine, Shanghai Children’s Medical Center, School of Medicine, Shanghai Jiao Tong University, Shanghai, China; ^2^ Department of Respiratory Medicine, Shanghai Children’s Medical Centre Affiliated to Shanghai Jiaotong University School of Medicine, Fujian Children’s Hospital, Fujian Maternity and Child Health Hospital, College of Clinical Medicine for Obstetrics & Gynecology And Pediatrics, Fujian Medical University, Fuzhou, Fujian, China; ^3^ Department of Respiratory Medicine, The Second Hospital of Jiaxing, Jiaxing, Zhejiang, China; ^4^ Department of Respiratory Medicine, Guizhou Provincial People’s Hospital, Shanghai Children’s Medical Center, Shanghai JiaoTong University School of Medicine, Guiyang, Guizhou, China; ^5^ Department of Respiratory Medicine, Shaoxing Central Hospital, Shaoxing, Zhejiang, China; ^6^ Department of Respiratory Medicine, Zhoupu Hospital Affiliated to Shanghai University of Medicine & Health Sciences, Shanghai, China; ^7^ Department of Respiratory Medicine, Ningbo Medical Center Lihuili Hospital, Ningbo, Zhejiang, China; ^8^ Department of Respiratory Medicine, Sanya Women and Children’s Hospital Affiliated to Hainan Medical College, Shanghai Children’s Medical Center, Sanya, Hainan, China; ^9^ Department of Respiratory Medicine, Linyi Maternal and Child Healthcare Hospital, Linyi, Shandong, China; ^10^ HongHe MCH (HongHe Hani and Yi Autonomous Prefecture Maternal and Child Health Hospital), Honghe, Yunnan, China; ^11^ Department of Respiratory Medicine, Shanghai Children’s Medical Center Pediatric Medical Complex (Pudong), Shanghai, China; ^12^ Pediatric Artificial Intelligence Clinical Application and Research Center, Shanghai Children’s Medical Center, Shanghai, China

**Keywords:** SARS-CoV-2 infection, allergic rhinitis, asthma, atopic dermatitis, incidence of symptoms, severity of symptoms

## Abstract

**Background:**

The relationship between allergic diseases and the adverse outcomes of severe acute respiratory syndrome coronavirus 2 (SARS-CoV-2) infection has been a subject of controversy. This study aimed to investigate the association between allergic diseases and the incidence and severity of symptoms in SARS-CoV-2 infection.

**Methods:**

Clinical data of individuals, including children and their parents, infected with SARS-CoV-2 from December 2022 to January 2023 in China were retrospectively analyzed. The data were collected through questionnaires. Statistical analysis, including chi-squared tests, nonparametric analysis, one-way ANOVA, and logistic regression analysis, was used to examine the relationship between allergic diseases, prior medication, and the symptoms of SARS-CoV-2 infection.

**Results:**

There were 3,517 adults and 3,372 children with SARS-CoV-2 infection included in the study. Fever was found to occur at similar rates in children (86.5%) and adults (86.8%). However, other symptoms related to respiratory issues (such as cough and sore throat), neurological symptoms (headache, loss of smell, and loss of taste), and systemic symptoms (muscle soreness and weakness) were observed more frequently in adults (*P* < 0.001). Additionally, adults exhibited higher overall symptom scores, indicating greater severity. Allergic diseases were found to be associated with the incidence of certain SARS-CoV-2 infection symptoms in both children and adults. Specifically, children with allergic rhinitis (AR) were observed to be more susceptible to upper respiratory symptoms (OR: 1.320, 95% CI: 1.081-1.611, *P* = 0.006), while asthma patients were found to be more susceptible to severe respiratory symptoms (OR: 1.736, 95% CI: 1.250-2.411, *P* = 0.001). Similar patterns were identified in adults. Furthermore, AR was also suggested to be a risk factor for symptom severity in both children (OR: 1.704, 95% CI: 1.314-2.209, *P* < 0.001) and adults (OR: 1.736, 95% CI: 1.250-2.411, *P* = 0.001). However, prior medication for allergic diseases did not exhibit a preventive effect on SARS-CoV-2 infection symptoms.

**Conclusions:**

Both children and adults with allergic diseases were found to be more prone to experiencing symptoms of SARS-CoV-2 infection, and these symptoms tended to be more severe.

## 1 Introduction

Severe acute respiratory syndrome coronavirus 2 (SARS-CoV-2) has been prevalent for over three years. Despite advances in vaccines, new drugs, improved medical and health policies, and doctors’ accumulated treatment experience, the rapid and widespread transmission of SARS-CoV-2 remains a significant concern. In December 2022, China lifted epidemic prevention policies related to SARS-CoV-2 infection, leading to a significant number of infections. Now, half a year later, as SARS-CoV-2 antibodies gradually diminish in patients, children and the elderly are confronting new health challenges. Individuals with comorbidities such as hypertension, diabetes, chronic obstructive pulmonary disease, obesity, and cardiovascular diseases may face an elevated risk of severe symptoms in corona virus disease 2019 (COVID-19) (1). This is compounded by increased levels of plasma proinflammatory cytokines, interferon type I (IFN-I), and angiotensin-converting enzyme 2 (ACE2) (2). Allergic rhinitis (AR) and asthma often co-occur as chronic respiratory comorbidities. While it is known that respiratory infections can exacerbate AR and asthma (3, 4), the impact of chronic respiratory comorbidities on patients with SARS-CoV-2 infection remains inconclusive. Some studies have suggested that AR and asthma act as protective factors against COVID-19 incidence, and asthma was associated with lower hospitalization of COVID-19 (5). Conversely, other literature reports have indicated that AR and asthma are not risk factors for COVID-19 aggravation (6, 7). Furthermore, certain cohort studies have proposed that patients with AR and asthma might have a heightened susceptibility to COVID-19 infection and severe symptoms (8, 9). Consequently, the relationship between allergic diseases and SARS-CoV-2 infection remains uncertain and necessitates further investigation with robust clinical data.

In this study, we gathered clinical data from children and adults with SARS-CoV-2 infection across various regions of China from December 2022 to January 2023. Our aim was to comprehensively examine the specific impact of allergic diseases such as AR, asthma, and atopic dermatitis (AD) on the incidence and severity of SARS-CoV-2 infection symptoms.

## 2 Methods

### 2.1 Study design and participants

This retrospective, multicenter, observational study was conducted across multiple hospitals in China from December 2022 to January 2023. Participating institutions included Shanghai Children’s Medical Center, Fujian Children’s Hospital, the Second Hospital of Jiaxing, Guizhou Provincial People’s Hospital, Shaoxing Central Hospital, Sanya Women and Children’s Hospital, Linyi Maternal and Child Healthcare Hospital, HongHe Hani and Yi Autonomous Prefecture Maternal and Child Health Hospital, among others. Trained pediatricians distributed questionnaires to children and their parents in respiratory outpatient and emergency departments. When they fill out the questionnaire, pediatricians provided guidance beside. The questionnaire took approximately 5-10 minutes to complete. Data was collected through the survey questionnaire backend.

The inclusion criteria including followings: (1) Children aged 0-18 years with SARS-CoV-2 infection from respiratory outpatient and emergency departments; (2) Adults (parents of the children) with SARS-CoV-2 infection; (3) Mild patients seeking outpatient treatment or observing at home. Exclusion criteria comprised any of the following: (1) Moderate to severe patients with SARS-CoV-2 infection; (2) Incomplete questionnaire survey.

This study received approval from the Institutional Review Board of Shanghai Children’s Medical Center, School of Medicine, Shanghai Jiao Tong University (approval number: SCMCIRB-K2023012-1). The need for informed consent was waived due to the retrospective nature of the study, and all patient information was handled anonymously.

### 2.2 Definition

In accordance with the “*Diagnosis, treatment, and prevention of severe acute respiratory syndrome coronavirus 2 infection in children: experts’ consensus statement (Fifth Edition) updated for the Omicron variant*” *(* 10) and the “*Diagnosis and treatment plan of SARS-CoV-2 Infection (Trial Version 10)*” (11) in China, the diagnosis of SARS-CoV-2 infection relied on a comprehensive analysis of epidemiological history, clinical manifestations, and laboratory tests. Confirmation of SARS-CoV-2 infection in this study was based on one of the following criteria: (1) Contact history with SARS-CoV-2 infected individuals and clinical manifestations of SARS-CoV-2 infection; (2) Positive result from a real-time RT-PCR assay of nasal or pharyngeal swabs; (3) Positive SARS-CoV-2 antigen test of nasal or pharyngeal swabs.

In accordance with the “*Guideline for the diagnosis and optimal management of asthma in children (2016)*” *(* 12), asthma diagnosis relied on respiratory symptoms (recurrent wheezing, coughing, shortness of breath, and chest tightness, often occurring or exacerbating at night and/or in the early morning), physical signs (wheezing sound), lung function tests (confirming variable expiratory airflow limitation), and exclusion of other diseases that could cause similar symptoms. At the beginning of the questionnaire, we set questions related to AR or AD, such as “Do you often have nose itching, running nose, nasal congestion, eczema, or urticaria in your daily life?” If the answer was yes, they would be evaluated and diagnosed by professional pediatric otolaryngologists and dermatologists, respectively, adhering to the “*Guidelines for the diagnosis and treatment of pediatric allergic rhinitis (2010, Chongqing)*” *(* 13) and the “*Consensus on the diagnosis and management of allergic diseases in children*” (14). As for these adults, their diagnosis of asthma, AR, and AD were diagnosed by professional adult pulmonary physicians, otolaryngologists and dermatologists. And they were classified based on the past medical records.

### 2.3 Data information

The data we collected from patients included their age, place of residence (divided into regions based on the Qinling Mountains-Huaihe Rivers as the dividing line between South and North China), gender, vaccine inoculation status, medications taken within one month, and more. Our primary inquiries revolved around SARS-CoV-2 infection and the diagnostic methods employed. For patients who were confirmed to be infected, we conducted detailed inquiries regarding the occurrence and severity of their symptoms. Additionally, we gathered information on the treatment administered and any adverse reactions to oral medications.

We provided a list of multiple symptoms (including fever, cough, sore throat, hoarseness, nasal congestion, runny nose, wheezing, weakness, headache, muscle soreness, vomiting, diarrhea, chest distress, shortness of breath, dyspnea, loss of smell, loss of taste, and convulsion) for patients to select from. We used the Visual Analog Scale (VAS) scoring system to assess the severity of each symptom, except for fever. The scoring ranged from 0 to 10, with “0” indicating “no symptoms” and “10” indicating “very severe symptoms”.

### 2.4 Statistics analyses

Data were analyzed using SPSS 23.0, and graphs were generated using GraphPad Prism 9.0. Categorical data were summarized with frequencies, percentages, and descriptive statistics. Firstly, the association between individual symptoms and allergic diseases was assessed using the chi-square test. Secondly, since the severity of individual symptoms did not follow a normal distribution, it was represented by median values (Q25, Q75). Differences in symptom severity among different types of allergic diseases were analyzed using the Mann-Whitney U test and Kruskal-Wallis (K-W, H) test. The overall severity score for all symptoms was calculated using means and standard deviations, as it conformed to the distribution of normal continuous data. One-way ANOVA was employed to assess the impact of different allergic diseases on the overall severity of symptoms, with *post hoc* comparisons using the LSD test. Thirdly, logistic regression analysis was conducted to determine the association between allergic diseases and SARS-CoV-2 infection symptoms, adjusting for age, vaccination status, region, and gender. Finally, logistic regression analysis was used to examine the association between previous medication and SARS-CoV-2 infection symptoms. A *P*-value less than 0.05 was considered statistically significant.

## 3 Results

### 3.1 Characteristics of the study population

As presented in **Table 1**, the questionnaire survey included a total of 3517 adults and 3372 children with SARS-CoV-2 infection. Fever and respiratory symptoms were the most common symptoms in both children and adults. The incidence of fever was similar between children (86.5%) and adults (86.8%), but other symptoms were more frequent in adults, including respiratory symptoms [cough (81.5% vs. 64.1%, *P*<0.001)], gastrointestinal symptoms [diarrhea (12.1% vs. 7.0%, *P*<0.001)], and systemic symptoms [weakness (55.9% vs. 20.0%, *P*<0.001) and muscle soreness (55.3% vs. 14.3%, *P*<0.001)]. Children received more treatments than adults, including oral medication (83.3% vs. 80.9%, *P*=0.010), intravenous rehydration (19.9% vs. 5.0%, *P*<0.001), hospitalization (4.5% vs. 0.6%, *P*<0.001), and oxygen inhalation (1.6% vs. 0.8%, *P*=0.004). Additionally, children experienced more adverse reactions to oral medications than adults, such as abdominal pain (2.0% vs. 1.3%, *P*=0.038) and vomiting (2.8% vs. 1.8%, *P*=0.014).

**Table 1:** Clinical characteristics of all study subjects.

Covariate	SARS-CoV-2 infection							
	Adults	Children		Allergic diseases in children				
	Total (n = 3517) (%)	Total (n = 3372) (%)	*P* value	Asthma (n = 932) (%)	Allergic rhinitis (n = 1252) (%)	Atopic dermatitis (n = 655) (%)	Without allergic diseases (n = 1604) (%)	*P* value
Gender			<0.001					**<0.001**
female	2898 (82.4)	1434 (42.5)		310 (33.3)	442 (35.3)	251 (38.3)	776 (48.4)	
male	619 (17.6)	1938 (57.5)		622 (66.7)	810 (64.7)	404 (61.7)	828 (51.6)	
Age								**<0.001**
0-2 years	NC	569 (16.9)		14 (1.5)	14 (1.1)	93 (14.2)	453 (28.2)	
3-6 years	NC	1059 (31.4)		305 (32.7)	377 (30.1)	228 (34.8)	496 (31.0)	
7-12 years	NC	1354 (40.1)		526 (56.5)	706 (56.4)	283 (43.2)	465 (29.0)	
13-18 years	NC	390 (11.6)		87 (9.3)	155 (12.4)	51 (7.8)	190 (11.8)	
Residence			0.35					**<0.001**
South	2299 (65.4)	2168 (64.3)		702 (75.3)	939 (75.0)	455 (69.5)	887 (55.3)	
North	1218 (34.6)	1204 (35.7)		230 (24.7)	313 (25.0)	200 (30.5)	717 (44.7)	
Diagnostic method							
nucleic acid testing	2053 (58.4)	345 (10.2)		89 (9.5)	118 (9.4)	52 (8.0)	713 (44.5)	
antigen dectection	732 (20.8)	1719 (51.0)		565 (60.6)	735 (58.7)	369 (56.3)	186 (11.6)	
symptoms	564 (16.0)	909 (27.0)		187 (20.1)	280 (22.4)	168 (25.6)	485 (30.2)	
history of exposure	168 (4.8)	399 (11.8)		91 (9.8)	119 (9.5)	66 (10.1)	220 (13.7)	
Past SARS-CoV-2 infection	NC	152 (4.5)		35 (3.8)	36 (2.9)	22 (3.4)	96 (6.0)	**<0.001**
Vaccination status	NC	1940 (57.5)		598 (64.2)	873 (69.7)	316 (48.2)	829 (51.7)	**<0.001**
Symptoms							
fever	3043 (86.5)	2960 (87.8)	0.117	808 (86.7)	1092 (87.2)	579 (88.4)	1420 (88.5)	0.484
cough	2867 (81.5)	2160 (64.1)	**<0.001**	610 (65.5)	821 (65.6)	411 (62.7)	1008 (62.8)	0.315
sore throat	1777 (50.5)	754 (22.4)	**<0.001**	203 (21.8)	319 (25.5)	133 (20.3)	343 (21.4)	**0.022**
hoarseness	935 (26.6)	447 (13.3)	**<0.001**	103 (11.1)	144 (11.5)	84 (12.8)	236 (14.7)	**0.021**
nasal congestion	1811 (51.5)	1281 (38.0)	**<0.001**	374 (40.1)	539 (43.1)	262 (40.0)	553 (34.5)	**<0.001**
runny nose	1482 (42.1)	1264 (37.5)	**<0.001**	378 (40.6)	505 (40.3)	252 (38.5)	573 (35.7)	**0.034**
wheezing	331 (9.4)	195 (5.8)	**<0.001**	61 (6.5)	59 (4.7)	42 (6.4)	104 (6.5)	0.169
weakness	1965 (55.9)	674 (20.0)	**<0.001**	218 (23.4)	298 (23.8)	155 (23.7)	275 (17.1)	**<0.001**
headache	1694 (48.2)	682 (20.2)	**<0.001**	236 (25.3)	345 (27.6)	150 (22.9)	246 (15.3)	**<0.001**
muscle soreness	1944 (55.3)	481 (14.3)	**<0.001**	156 (16.7)	229 (18.3)	107 (16.3)	190 (11.8)	**<0.001**
Vomiting	276 (7.8)	390 (11.6)	**<0.001**	110 (11.8)	166 (13.3)	99 (15.1)	165 (10.3)	**0.007**
diarrhea	425 (12.1)	237 (7.0)	**<0.001**	56 (6.0)	76 (6.1)	54 (8.2)	118 (7.4)	0.181
chest distress	477 (13.6)	52 (1.5)	**<0.001**	16 (1.7)	27 (2.2)	6 (0.9)	22 (1.4)	0.170
shortness of breath	332 (9.4)	103 (3.1)	**<0.001**	32 (3.4)	40 (3.2)	23 (3.5)	46 (2.9)	0.816
dyspnea	180 (5.1)	64 (1.9)	**<0.001**	20 (2.1)	22 (1.8)	11 (1.7)	32 (2.0)	0.878
loss of smell	820 (23.3)	100 (3.0)	**<0.001**	28 (3.0)	44 (3.5)	18 (2.7)	43 (2.7)	0.605
loss of taste	1014 (28.8)	143 (4.2)	**<0.001**	38 (4.1)	58 (4.6)	26 (4.0)	61 (3.8)	0.733
convulsion	11 (0.3)	21 (0.6)	0.059	5 (0.5)	6 (0.5)	8 (1.2)	9 (0.6)	0.225
other symptoms	60 (1.7)	55 (1.6)	0.808	10 (1.1)	19 (1.5)	17 (2.6)	25 (1.6)	0.119
no obvious symptoms	82 (2.3)	221 (6.6)	**<0.001**	70 (7.5)	91 (7.3)	44 (6.7)	91 (5.7)	0.227
Diagnosis of pneumonia	NC	183 (5.4)		28 (3.0)	43 (3.4)	26 (4.0)	119 (7.4)	**<0.001**
hospitalization	NC	96 (52.5)		15 (53.6)	19 (44.2)	16 (61.5)	62 (52.1)	0.567
oxygen inhalation	NC	25 (13.7)		3 (10.7)	2 (4.7)	1 (3.8)	20 (1.7)	0.091
Treatment							
oral medicine	2846 (80.9)	2809 (83.3)	**0.010**	783 (84.0)	1035 (82.7)	552 (84.3)	1335 (83.2)	0.769
antipyretic	2147 (75.4)	2361 (84.1)	**<0.001**	646 (82.5)	871 (84.2)	468 (84.8)	1124 (84.2)	0.663
antitussive	1091 (38.3)	1093 (38.9)	0.656	346 (44.2)	428 (41.4)	197 (35.7)	498 (37.3)	**0.002**
coldrex	886 (31.1)	612 (21.8)	**<0.001**	157 (20.1)	190 (18.4)	106 (19.2)	324 (24.3)	**<0.001**
antiviral drugs	319 (11.2)	397 (14.1)	**<0.001**	95 (12.1)	138 (13.3)	78 (14.1)	200 (15.0)	0.303
antibiotic	440 (15.5)	400 (14.2)	0.197	144 (18.4)	178 (17.2)	86 (15.6)	153 (11.5)	**<0.001**
Chinese patent drug	549 (19.3)	483 (17.2)	**0.041**	134 (17.1)	191 (18.5)	91 (16.5)	214 (16.0)	0.466
antidiarrheal	27 (0.9)	58 (2.1)	**0.001**	11 (1.4)	17 (1.6)	11 (2.0)	32 (2.4)	0.368
other oral medicine	121 (4.3)	153 (5.4)	**0.036**	62 (7.9)	75 (7.2)	36 (6.5)	56 (4.2)	**0.002**
nebulization therapy	177 (5.0)	671 (19.9)	**<0.001**	274 (35.0)	275 (22.0)	146 (22.3)	285 (17.8)	**<0.001**
intravenous rehydration	111 (3.2)	212 (6.3)	**<0.001**	37 (4.7)	52 (4.2)	44 (6.7)	131 (8.2)	**<0.001**
oxygen inhalation	29 (0.8)	53 (1.6)	**0.004**	11 (1.4)	12 (1.0)	9 (1.4)	34 (2.1)	0.059
hospitalization	20 (0.6)	153 (4.5)	**<0.001**	24 (3.1)	34 (2.7)	30 (4.6)	93 (5.8)	**<0.001**
other	73 (2.1)	10 (3.2)	**0.004**	27 (3.4)	51 (4.1)	20 (3.1)	39 (2.4)	0.090
Adverse reactions of oral medicine							
diarrhea	173 (6.1)	139 (4.9)	0.063	31 (4.0)	31 (3.0)	28 (5.1)	75 (5.6)	**0.016**
abdominal pain	36 (1.3)	55 (2.0)	**0.038**	18 (2.3)	23 (2.2)	10 (1.8)	24 (1.8)	0.808
vomiting	52 (1.8)	79 (2.8)	**0.014**	20 (2.6)	34 (3.3)	15 (2.7)	34 (2.5)	0.705
poor appetite	348 (12.2)	329 (11.7)	0.654	88 (11.2)	130 (12.6)	80 (14.5)	133 (10.0)	**0.029**
erythra	40 (1.4)	53 (1.9)	0.155	20 (2.6)	17 (1.6)	23 (4.2)	15 (1.1)	**<0.001**
other	41 (1.4)	40 (1.4)	0.958	4 (0.5)	10 (1.0)	7 (1.3)	19 (1.4)	0.245
Underlying disease								
asthma	68 (1.9)	932 (27.6)	**<0.001**		670 (53.5)	275 (42.0)		
allergic rhinitis	682 (19.4)	1252 (37.1)	**<0.001**	670 (71.9)		363 (55.4)		
allergic dermatitis	181 (5.1)	655 (19.4)	**<0.001**	275 (29.5)	363 (29.0)			
tracheal stenosis	NC	32 (0.95)		17 (1.8)	19 (1.5)	5 (0.8)	9 (0.6)	**0.012**
bronchitis obliterans	NC	25 (0.74)		7 (0.8)	10 (0.8)	6 (0.9)	11 (0.7)	0.950
bronchiectasia	NC	34 (1.0)		13 (1.4)	20 (1.6)	6 (0.9)	10 (0.6)	0.068
other system diseases	202 (5.7)	194 (5.8)	0.986	15 (1.6)	108 (8.6)	46 (7.0)	54 (3.4)	**<0.001**

SARS-CoV-2, Severe acute respiratory syndrome coronavirus 2; Bold indicates statistical significance, P <0.05; “NC” means not counted.

Among adults, there were 68 patients with asthma, 682 with AR, and 181 with AD. In children, there were 932 with asthma, 1252 with AR, and 655 with AD. We further compared the clinical characteristics of allergic and non-allergic children and found that the proportion of boys was higher than girls (*P*<0.001), and more patients resided in the southern regions of China (*P*<0.001). Additionally, the ratio of children aged 3-12 years was higher than that of younger children (aged 0-2 years) and older children (aged 3-12 years) (*P*<0.001).

### 3.2 Association between allergic diseases and the incidence of symptoms

**Table 1** revealed that allergic diseases were associated with higher incidences of certain symptoms, including sore throat (*P* = 0.022), hoarseness (*P* = 0.021), nasal congestion (*P* < 0.001), runny nose (*P* = 0.034), weakness (*P* < 0.001), headache (*P* < 0.001), muscle soreness (*P* < 0.001), and vomiting (*P* = 0.007). Additionally, children with allergic diseases received more nebulization therapy. However, the proportions of pneumonia and hospitalization were higher in children without allergic diseases compared to children with allergic diseases.

### 3.3 Association between allergic diseases and the severity of symptoms

**Figure 1:**
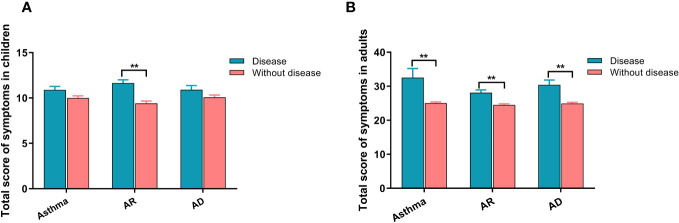
The relationship between allergic diseases and symptom severity in SARS-CoV-2 infection for both children and adults. **(A)** Severity of symptoms in children. **(B)** Severity of symptoms in adults. AR, allergic rhinitis; AD, atopic dermatitis. ** indicates *P <*0.01.

**Table 2** presents the analysis of the relationship between different allergic diseases and the severity of each symptom of SARS-CoV-2 infection. The results indicated that children with allergic diseases, whether they had one type (asthma, AR, or AD), two types, or all three types of allergies, experienced more severe symptoms than those without any allergies. This increased severity was observed for symptoms such as cough, sore throat, hoarseness, nasal congestion, runny nose, headache, weakness, muscle soreness, and vomiting. Similarly, adults with allergic diseases also displayed increased severity in most symptoms and were more susceptible to weakness, muscle soreness, wheezing, shortness of breath, loss of smell, loss of taste, and dyspnea. Subsequently, we calculated the total score of all symptoms for each patient and found that both children (**Figure 1A**) and adults (**Figure 1B**) with allergic diseases had higher scores than those without allergies, with adults having higher scores than children.

**Table 2:** The association between allergies on the severity of symptoms.

Symptoms	Children							Adults				
	no allergy	asthma	AR	AD	2 types of allergy	3 types of allergy	*P* value	no allergy	asthma	AR	AD	*P* value
cough	2 (0,4)	2 (0,4)^*^	2 (0,4)^*^	2 (0,4)	2 (0,4)	2 (0,4)	**0.014**	3 (1,5)	4 (3,6.75)^*^	4 (2,6)	4 (2,6)^*^	**<0.001**
sore throat	0 (0,0)	0 (0,0)	0 (0,1)^*^	0 (0,0)	0 (0,0)	0 (0,0)	**0.049**	0 (0,4)	0 (0,4)	2 (0,4)	0 (0,5)	0.581
hoarseness	0 (0,0)	0 (0,0)^*^	0 (0,0)^*^	0 (0,0)	0 (0,0)	0(0,0)	0.054	0 (0,1)	0 (0,0)	0 (0,2)	0 (0,2)	0.442
nasal congestion	0 (0,2)	0 (0,3)	0 (0,4)^*^	0 (0,3)	0 (0,4)^*^	0 (0,4)^*^	**<0.001**	0 (0,4)	1.5 (0,4)	2 (0,5)^*^	2 (0,4)	**0.002**
runny nose	0 (0,2)	0 (0,2)^*^	0 (0,3)^*^	0 (0,2)	0 (0,2)	0 (0,3)	**0.017**	0 (0,3)	0.5 (0,4)	0 (0,4)^*^	1 (0,4)^*^	**<0.001**
wheezing	0 (0,0)	0 (0,0)	0 (0,0)^*^	0 (0,0)	0 (0,0)	0 (0,0)	0.208	0 (0,0)	0 (0,4)^*^	0 (0,0)	0 (0,0)	**<0.001**
weakness	0 (0,0)	0 (0,0)^*^	0 (0,0)^*^	0 (0,0)^*^	0 (0,0)	0 (0,2)^*^	**<0.001**	2 (0,5)	4 (0,6)	3 (0,6)^*^	4 (0,6)^*^	**<0.001**
headache	0 (0,0)	0 (0,0)^*^	0 (0,4)^*^	0 (0,0)^*^	0 (0,1)^*^	0 (0,2)^*^	**<0.001**	0 (0,4)	2 (0,6)	2 (0,6)^*^	2 (0,6)^*^	**<0.001**
muscle soreness	0 (0,0)	0 (0,0)^*^	0 (0,0)^*^	0 (0,0)	0 (0,0)^*^	0 (0,0)^*^	**<0.001**	2 (0,6)	4 (0,6)^*^	4 (0,6)^*^	4 (0,6)^*^	**<0.001**
vomiting	0 (0,0)	0 (0,0)	0 (0,0)^*^	0(0,0)^*^	0 (0,0)	0 (0,0)	**0.012**	0 (0,0)	0 (0,0)	0 (0,0)	0 (0,0)	0.185
diarrhea	0 (0,0)	0 (0,0)	0 (0,0)	0 (0,0)	0 (0,0)	0 (0,0)	0.352	0 (0,0)	0 (0,0)	0 (0,0)	0 (0,0)	0.435
chest distress	0 (0,0)	0 (0,0)	0 (0,0)^*^	0 (0,0)	0 (0,0)	0 (0,0)	0.268	0 (0,0)	0 (0,0)	0 (0,0)	0 (0,0)	**0.019**
shortness of breath	0 (0,0)	0 (0,0)	0 (0,0)	0 (0,0)	0 (0,0)	0 (0,0)	0.941	0 (0,0)	0 (0,0)^*^	0 (0,0)	0 (0,0)	**0.005**
dyspnea	0 (0,0)	0 (0,0)	0 (0,0)	0 (0,0)	0 (0,0)	0 (0,0)	0.865	0 (0,0)	0 (0,0)^*^	0 (0,0)	0 (0,0)	**<0.001**
loss of smell	0 (0,0)	0 (0,0)	0 (0,0)	0 (0,0)	0 (0,0)	0 (0,0)	0.877	0 (0,0)	0 (0,4)	0 (0,2)	0 (0,4)^*^	**<0.001**
loss of taste	0 (0,0)	0 (0,0)	0 (0,0)	0 (0,0)	0 (0,0)	0 (0,0)	0.944	0 (0,2)	0 (0,4)	0 (0,3)^*^	0 (0,4.5)^*^	**0.002**
convulsion	0 (0,0)	0 (0,0)	0 (0,0)	0 (0,0)	0 (0,0)	0 (0,0)	0.586	0 (0,0)	0 (0,0)	0 (0,0)	0 (0,0)	0.261

AR, Allergic rhinitis; AD, Atopic dermatitis; Bold indicates statistical significance among multiple groups in each symptom; *indicates P <0.05 compared with no allergy in each symptom.

### 3.4 Potentially affecting of allergic diseases on the incidence of symptoms

Based on clinical manifestations, all symptoms were classified into five categories: upper respiratory symptoms (cough, sore throat, hoarseness, nasal congestion and runny nose), severe respiratory symptoms (wheezing, chest distress, shortness of breath, and dyspnea), systemic symptoms (fever, weakness, and muscle soreness), neurological symptoms (headache, loss of smell, loss of taste, and convulsion), and gastrointestinal symptoms (vomiting and diarrhea).

As shown in **Table 3**, individuals with asthma, both children (OR: 1.736, 95% CI: 1.250-2.411, *P* = 0.001) and adults (OR: 3.007, 95% CI: 1.831-4.938, *P* < 0.001), were more likely to experience severe respiratory symptoms compared to those without allergies. Children and adults with AR were more prone to upper respiratory [(OR: 1.320, 95% CI: 1.081-1.611, *P* = 0.006); (OR: 1.427, 95% CI: 1.040-1.956, *P* = 0.027)] and neurological [(OR: 1.317, 95% CI: 1.084-1.602, *P* = 0.006); (OR: 1.448, 95% CI: 1.210-1.734, *P* < 0.001)] symptoms. Additionally, adults with AR were also more susceptible to gastrointestinal symptoms (OR: 1.282, 95% CI: 1.033-1.591, *P* = 0.024). However, allergies appeared to have no significant impact on the incidence of systemic symptoms.

**Table 3:** Potentially affecting of allergic diseases on the incidence of symptoms.

Outcomes	Children				Adults			
	Crude OR (95%CI)	*P* value	Adjusted OR (95%CI)	*P* value	Crude OR (95%CI)	*P* value	Adjusted OR (95%CI)	*P* value
The incidence of upper respiratory symptoms								
Asthma	0.874 (0.716, 1.067)	0.187	0.990 (0.806, 1.216)	0.923	1.205 (0.516, 2.811)	0.667	1.096 (0.466, 2.577)	0.833
AR	1.270 (1.051, 1.534)	**0.013**	1.320 (1.081, 1.611)	**0.006**	1.442 (1.056, 1.970)	**0.021**	1.427 (1.040, 1.956)	**0.027**
AD	0.816 (0.668, 0.998)	**0.048**	0.895 (0.728, 1.099)	0.289	1.994 (1.041, 3.819)	**0.037**	1.790 (0.931, 3.441)	**0.081**
The incidence of severe respiratory symptoms								
Asthma	1.414 (1.041, 1.922)	**0.027**	1.736 (1.250, 2.411)	**0.001**	3.114 (1.911, 5.074)	**<0.001**	3.007 (1.831, 4.938)	**<0.001**
AR	0.678 (0.501, 0.917)	**0.012**	0.863 (0.621, 1.200)	0.382	1.180 (0.963, 1.444)	0.110	1.175 (0.957, 1.443)	0.124
AD	1.106 (0.805, 1.518)	0.534	1.068 (0.774, 1.473)	0.688	1.014 (0.697, 1.473)	0.944	0.940 (0.645, 1.370)	0.747
The incidence of systemic symptoms								
Asthma	0.911 (0.697, 1.191)	0.495	0.977 (0.745, 1.280)	0.865	1.525 (0.474, 4.901)	0.479	1.589 (0.492, 5.133)	0.439
AR	0.966 (0.751, 1.242)	0.785	1.076 (0.829, 1.397)	0.582	1.263 (0.872, 1.828)	0.217	1.289 (0.888, 1.871)	0.181
AD	1.121 (0.842, 1.491)	0.433	1.100 (0.824, 1.470)	0.517	1.522 (0.736, 3.144)	0.257	1.489 (0.718, 3.085)	0.284
The incidence of neurological symptoms								
Asthma	1.152 (0.947, 1.400)	0.156	1.133 (0.925, 1.388)	0.229	1.405 (0.845, 2.337)	0.19	1.423 (0.850, 2.380)	0.179
AR	1.751 (1.457, 2.105)	**<0.001**	1.317 (1.084, 1.602)	**0.006**	1.421 (1.190, 1.696)	**<0.001**	1.448 (1.210, 1.734)	**<0.001**
AD	1.045 (0.852, 1.281)	0.674	1.257 (1.013, 1.560)	**0.038**	1.882 (1.346, 2.631)	**<0.001**	1.816 (1.295, 2.547)	**0.001**
The incidence of gastrointestinal symptoms								
Asthma	0.902 (0.717, 1.135)	0.379	0.926 (0.730, 1.175)	0.528	1.185 (0.642, 2.188)	0.587	1.217 (0.657, 2.255)	0.533
AR	1.085 (0.878, 1.341)	0.447	1.224 (0.975, 1.536)	0.082	1.260 (1.018, 1.561)	**0.034**	1.282 (1.033, 1.591)	**0.024**
AD	1.467 (1.176, 1.829)	**0.001**	1.392 (1.112, 1.741)	**0.004**	0.996 (0.664, 1.494)	0.985	0.978 (0.651, 1.469)	0.914

Adjusted: Models of Children were adjusted by age (0-2, 3-6, 7-12, 13-18 years old), vaccination status (yes or no), regions (South or Nouth of China) and gender. Models of Adults were adjusted by age (19-30, 31-50, ≥51 years old), regions (South or Nouth of China) and gender. AR, allergic rhinitis; AD, atopic dermatitis; CI, confidence interval; OR, odds risk. Bold indicates statistical significance.

### 3.5 Potentially affecting of allergic diseases on the severity of symptoms

The severity of symptoms was divided into four grades based on the total score of all symptoms: grade I (asymptomatic), grade II (1-10), grade III (11-30), and grade IV (≥ 31). Subgroup analysis in **Table 4** revealed that AR was a risk factor for the severity of symptoms in both children (OR: 1.712, 95% CI: 1.161-2.524, *P* = 0.007) and adults (OR: 2.013, 95% CI: 2.273-3.184, *P* = 0.003). However, the sample sizes of asthma and AD in adults were too small to calculate an accurate OR (95% CI), and therefore, this result was not recorded.

**Table 4:** Potentially affecting of allergic diseases on the severity of symptoms.

Outcomes	Children				Adults			
	Crude OR (95%CI)	*P* value	Adjusted OR (95%CI)	*P* value	Crude OR (95%CI)	*P* value	Adjusted OR (95%CI)	*P* value
**0 score (grade I)**	1 (1,1)		1 (1,1)		1 (1,1)		1 (1,1)	
1-10 scores (grade II)								
Asthma	1.139 (0.889, 1.459)	0.302	1.224 (0.951, 1.577)	0.117	NR		NR	
AR	1.135 (0.901, 1.430)	0.282	1.144 (0.898, 1.457)	0.275	1.220 (0.763, 1.953)	0.406	1.160 (0.722, 1.862)	0.539
AD	1.067 (0.828, 1.374)	0.618	1.149 (0.889, 1.485)	0.288	NR		NR	
11-30 scores (grade III)								
Asthma	1.068 (0.818, 1.395)	0.629	1.160 (0.883, 1.525)	0.286	NR		NR	
AR	1.746 (1.363, 2.236)	**<0.001**	1.704 (1.314, 2.209)	**<0.001**	1.722 (1.097, 2.705)	**0.018**	1.633 (1.035, 2.575)	**0.035**
AD	0.979 (0.743, 1.291)	0.881	1.080 (0.816, 1.430)	0.591	NR		NR	
≥31 scores (grade IV)								
Asthma	0.897 (0.604, 1.331)	0.588	1.076 (0.712, 1.626)	0.728	NR		NR	
AR	1.822 (1.272, 2.612)	**0.001**	1.712 (1.161, 2.524)	**0.007**	2.076 (1.320, 3.265)	**0.002**	2.013 (1.273, 3.184)	**0.003**
AD	1.291 (0.876, 1.903)	0.197	1.615 (1.080, 2.414)	**0.020**	NR		NR	

Adjusted: Models of Children were adjusted by age (0-2, 3-6, 7-12, 13-18 years old), vaccination status (yes or no), regions (South or Nouth of China) and gender. Models of Adults were adjusted by age (19-35, 36-50, ≥51 years old), regions (South or Nouth of China) and gender. AR, allergic rhinitis; AD, atopic dermatitis. CI, confidence interval; OR, odds risk. NR, not recorded. Bold indicates statistical significance.

### 3.6 Association between previous medication and the symptoms of SARS-CoV-2 infection in children

**Figure 2:**
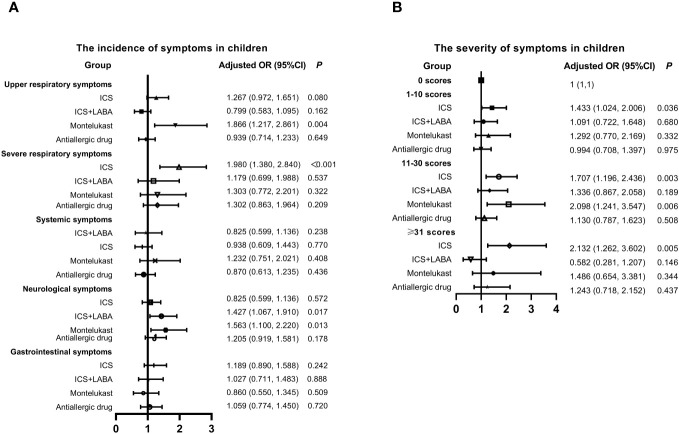
Relationship between Previous Medication, Symptom Incidence, and Severity in Children with SARS-CoV-2 Infection. **(A)** Incidence of symptoms. **(B)** Severity of symptoms. Adjusted: Models of Children were adjusted by age (0-2, 3-6, 7-12, 13-18 years old), vaccination status (yes or no), regions (South or Nouth of China) and gender. ICS, inhaled corticosteroids; LABA, long acting beta agonist; CI, confidence interval; OR, odds risk.

Four main long-term medication types commonly used by children for controlling symptoms of AR or asthma were identified, including inhaled corticosteroids (ICS) (budesonide, beclomethasone dipropionate, or fluticasone propionate), ICS+long-acting beta-agonist (LABA) (budesonide/formoterol or salmeterol/fluticasone), leukotriene receptor antagonist (montelukast), and antiallergic drugs. Subsequently, the relationship between previous medication within one month, incidence, and the severity of symptoms was analyzed in **Figure 2**. Montelukast was identified as a risk factor for the incidence of upper respiratory (OR: 1.866, 95% CI: 1.217-2.861, *P* = 0.004) and neurological (OR: 1.563, 95% CI: 1.100-2.220, *P* = 0.013) symptoms. ICS was associated with an increased incidence of severe respiratory symptoms (OR: 1.980, 95% CI: 1.380-2.840, P=0.003). Moreover, individuals who used montelukast (OR: 1.707, 95% CI: 1.196-2.436, *P* < 0.001) and ICS (OR: 2.098, 95% CI: 1.241-3.547, *P* = 0.006) experienced more severe symptoms. In contrast, there was no significant correlation between antiallergic drugs and the occurrence and severity of symptoms.

### 3.7 Association between vaccination status and the symptoms of SARS-CoV-2 infection in children

**Figure 3:**
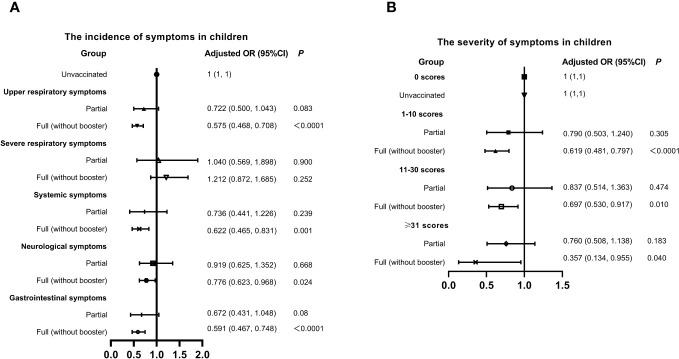
Relationship between Vaccination status, Symptom Incidence, and Severity in Children with SARS-CoV-2 Infection. **(A)** Incidence of symptoms. **(B)** Severity of symptoms. Adjusted: Models of Children were adjusted by age (0-2, 3-6, 7-12, 13-18 years old), regions (South or Nouth of China), allergy (yes or no) and gender. CI, confidence interval; OR, odds risk.

China promoted vaccination actively, but the breakthrough infections had emerged in many vaccine recipients. Here, we evaluated the association of vaccination and the symptoms of infected children in **Figure 3**. The overall vaccination rate (full inactivated vaccine) was 57.53% in children, of which only 9.02% got partial vaccination and 90.11% got full (without booster) vaccination. Full (without booster) vaccination seemed to reduce the incidence of symptoms SARS-CoV-2 infection except the severe respiratory and gastrointestinal symptoms. Similarly, the probability of experiencing severe symptoms was lower in children got full vaccination, compared to those unvaccinated. The population of booster vaccination was too small, and was not included in the statistics.

## 4 Discussion

In this study, we analyzed the clinical symptoms of 3372 children and 3517 adults who had contracted SARS-CoV-2 infection. Among them, the proportion of individuals with allergic diseases, especially asthma, in children was higher than the normal population. This difference can be attributed to the fact that our study included two distinct groups: the respiratory outpatient and emergency population. And the former including a portion of asthma follow-up population. This allowed for a more robust comparison between allergic and non-allergic patients in terms of their SARS-CoV-2 infection symptoms. In addition, among the children with SARS-CoV-2 infection, the population of boys with asthma were higher than girls, which was consistent with the gender distribution of asthma children in previous research (15, 16). The difference may be due to the sex hormones, genetic and epigenetic variations, social and environmental factors and so on (17, 18). However, the relationship between gender differences of asthma and SARS-CoV-2 infection are still controversial. Moreover, the regional differences in the population of asthma children, may be attributed to the climate and environment. In south of China, the moist air, mold, pollen, cockroaches, low latitude and low temperature in winter are all related to asthma (19–21). On the contrary, the indoor heating in northern China avoids the stimulation of low temperature significantly.

There are some papers shows controversy about the association of allergic diseases and SARS-CoV-2 infection. Some studies reported that allergic diseases have no obvious correlation with COVID-19, and others showed that Patients with allergic diseases may have a lower risk of COVID-19 (5–7). However, our findings revealed a strong association between allergic diseases and the incidence and severity of SARS-CoV-2 infection symptoms. Further investigation indicated that individuals with allergic diseases were more susceptible to developing symptoms upon SARS-CoV-2 infection, particularly respiratory and neurological symptoms. Furthermore, once clinical symptoms manifested, patients with asthma, AR, or AD tended to experience more severe clinical symptoms following SARS-CoV-2 infection compared to those without allergies. This aligns with similar results from a national cohort study in South Korea (8), which found that AR and asthma conferred a higher risk of susceptibility to SARS-CoV-2 infection and severe clinical outcomes of COVID-19. Additionally, a prospective cohort study demonstrated that a history of allergic disease posed a risk factor for asthma-like symptoms following COVID-19 hospitalization, and COVID-19 presentations were more severe in the asthma-like group (9). Those controversial results may be attributed to diet, lifestyle, geographical differences, ethnicity, exposed viral load, and quality of health care (22), and also the differences in asthma endotypes (23).

In this study, we fore, it is crucial to explore the mechanisms underlying how allergic diseases exacerbate symptoms of SARS-CoV-2 infection. The severity of symptoms in SARS-CoV-2 infection may be linked to viral load within host cells. The transmembrane protease serine 2 (TMPRSS2) serves as the receptor facilitating SARS-CoV-2 entry into cells by cleaving the spike protein into two subunits (24). TMPRSS2 exhibits high expression levels in human primary bronchial epithelial cells and the nasal lining and may be jointly regulated by airway inflammation and environmental factors, as it shows heightened expression in asthma and AR (25, 26). Consequently, this could increase the likelihood of SARS-CoV-2 entering the airways. Furthermore, researchers have observed higher levels of TMPRSS2 and ACE2 in the lesional skin of AD patients (25), potentially explaining their susceptibility to SARS-CoV-2 and the likelihood of experiencing more severe symptoms upon infection. Additionally, allergic disease patients may exhibit inefficient antiviral defense mechanisms, resulting in delayed antiviral responses by interfering with plasmacytoid dendritic and epithelial cells’ production of IFN-α/β/λ (27). The high-affinity IgE receptor (FcϵRI) has also been linked to reductions in virus-induced IFN-α (28, 29), as anti-IgE monoclonal antibody (Omalizumab) treatment has been shown to reduce FcϵRI expression and potentially enhance antiviral responses (30). In summary, these findings provide a compelling physiological rationale for discovering that allergic diseases act as risk factors for both the incidence and severity of SARS-CoV-2 infection symptoms.

It’s intriguing that our findings showed that children who had previously received treatment with ICS and montelukast were more susceptible to SARS-CoV-2 infection, and their symptoms were more severe. This contrasts with some reported results. ICS is a fundamental component of allergic disease treatment, often used as anti-inflammatory control therapy (31). A UK study indicated that the use of ICS in individuals at higher risk of complications due to COVID-19 could shorten the time to recovery and potentially reduce hospital admissions or fatalities (32). Izquierdo et al (33), in their analysis of clinical data from 71,182 asthma patients, found that a significantly higher percentage of non-hospitalized patients used ICS compared to asthma patients who required hospitalization due to COVID-19. Furthermore, there have been reports suggesting that montelukast can effectively reduce the incidence rate and mortality associated with “long-term COVID-19” (34), as well as the occurrence of clinical deterioration in hospitalized COVID-19 patients (35). These findings suggest that ICS and montelukast may have potential preventive effects against SARS-CoV-2 infection. However, our results contradict the above findings. This discrepancy could be attributed to two possible factors. First, children with well-controlled asthma or AR may discontinue their medication. This non-standard use of medication might result in poor efficacy of ICS and montelukast when confronted with SARS-CoV-2. The other reason could be that children who received medication within one month might represent patients with more severe allergic diseases. The use of medication often signifies the severity of allergies, which in turn might imply a higher likelihood of experiencing severe clinical symptoms following SARS-CoV-2 infection.

In this study, although it was found that vaccination could not avoid SARS-CoV-2 infection completely, the full (without booster) vaccination had a protective effect on the incidence and severity of symptoms with SARS-CoV-2 infection, which was consistent with some researches (36–38). However, different types of vaccines (39), age and frequency of vaccination (38), and occasion of vaccination (40) may affect the effectiveness, which needs more detailed research in the future. Another intriguing finding from our study is the potential relationship between age and the symptoms of SARS-CoV-2 infection. Adults exhibited more severe symptoms of SARS-CoV-2 infection compared to children, which aligns with similar findings in other studies. Children with COVID-19 generally experience a milder clinical course with lower rates of severe morbidity and mortality compared to adults (41, 42). This could be linked to the increased expression of ACE2 in the nasal epithelium with age (43). Additionally, the reduced exposure of children to public places and schools during the epidemic might also contribute to their milder symptoms.

This study has several limitations that should be acknowledged. Firstly, the data collected in this study may be subject to some degree of bias as it relied on a questionnaire survey of parents. Parents had to judge the occurrence and severity of symptoms in children based on their own subjective observations, and some young children may not be able to accurately express their discomfort. Secondly, the data was gathered from outpatient and emergency department visits in hospitals, which mainly included mild cases. Therefore, it may not fully represent the entire population with SARS-CoV-2 infection, especially severe cases. Thirdly, half of the children were from respiratory outpatient department, so the proportion of asthmatic children in this article study is higher than the morbidity of asthma in children. In addition, asthma is a heterogeneous disease, we did not consider the impact of factors such as weight, environment, and different phenotypes on the results.

Despite these limitations, this study is the first large-scale investigation in China to assess the relationship between allergic diseases and the symptoms of SARS-CoV-2 infection in both children and adults. The notable strengths of our study include its substantial sample size and the inclusion of data from both children and adults (3372 children and 3517 adults). Consequently, our study provides robust evidence that allergic diseases are linked to the incidence and severity of symptoms associated with SARS-CoV-2 infection.

In summary, individuals with allergic diseases might be more susceptible to experiencing symptoms when infected with SARS-CoV-2. Moreover, if SARS-CoV-2 establishes clinical manifestations in patients with allergic diseases, the risk of severe symptoms is elevated, with this risk being even more pronounced in adults compared to children.

## Data availability statement

The raw data supporting the conclusions of this article will be made available by the authors, without undue reservation.

## Ethics statement

The studies involving humans were approved by Institutional Review Board of Shanghai Children’s Medical Center, School of Medicine, Shanghai Jiao Tong University (approval number: SCMCIRB-K2023012-1). The studies were conducted in accordance with the local legislation and institutional requirements. Written informed consent for participation was not required from the participants or the participants’ legal guardians/next of kin in accordance with the national legislation and institutional requirements.

## Author contributions

HZ: Formal analysis, Visualization, Writing – original draft. JL: Formal analysis, Visualization, Writing – review & editing. JW: Formal analysis, Visualization, Writing – review & editing. JZ: Data curation, Investigation, Writing – review & editing. LZ: Data curation, Investigation, Writing – review & editing. SY: Data curation, Investigation, Writing – review & editing. JiaC: Data curation, Investigation, Writing – review & editing. QT: Investigation, Writing – review & editing. AZ: Investigation, Writing – review & editing. YC: Investigation, Writing – review & editing. XX: Investigation, Writing – review & editing. HD: Investigation, Writing – review & editing. HS: Investigation, Writing – review & editing. XH: Funding acquisition, Investigation, Writing – review & editing. DX: Supervision, Writing – review & editing. JC: Visualization, Writing – review & editing. FH: Visualization, Writing – review & editing. YY: Conceptualization, Supervision, Funding acquisition, Writing – review & editing.
